# Associations between Plasma Branched Chain Amino Acids and Health Biomarkers in Response to Resistance Exercise Training Across Age

**DOI:** 10.3390/nu12103029

**Published:** 2020-10-02

**Authors:** Mariwan H. Sayda, Bethan E. Phillips, John P. Williams, Paul L. Greenhaff, Daniel J. Wilkinson, Ken Smith, Philip J. Atherton

**Affiliations:** 1 MRC-Versus Arthritis Centre for Musculoskeletal Ageing Research, University of Nottingham, Nottingham NG7 2RD, UK; mariwan.sayda@nottingham.ac.uk (M.H.S.); Beth.Phillips@nottingham.ac.uk (B.E.P.); john.williams7@nottingham.ac.uk (J.P.W.); paul.Greenhaff@nottingham.ac.uk (P.L.G.); Daniel.Wilkinson@nottingham.ac.uk (D.J.W.); ken.smith@nottingham.ac.uk (K.S.); 2 The National Centre for Sport and Exercise Medicine—East Midlands, University of Nottingham, Nottingham NG7 2UH, UK; 3 NIHR Biomedical Research Centre, University of Nottingham, Nottingham NG7 2UH, UK

**Keywords:** branched chain amino acids, health, resistance exercise, ageing

## Abstract

Leucine, isoleucine and valine (i.e., the branched chain amino acids, BCAA) play a key role in the support of tissue protein regulation and can be mobilized as energy substrates during times of starvation. However, positive relationships exist between elevated levels of BCAA and insulin resistance (IR). Thus, we sought to investigate the links between fasting plasma BCAA following a progressive resistance exercise training (RET) programme, an intervention known to improve metabolic health. Fasting plasma BCAA were quantified in adults (young: 18–28 y, *n* = 8; middle-aged: 45–55 y, *n* = 9; older: 65–75 y, *n* = 15; BMI: 23–28 kg/m^2^, both males and females (~50:50), in a cross-sectional, intervention study. Participants underwent 20-weeks whole-body RET. Measurements of body composition, muscle strength (1-RM) and metabolic health biomarkers (e.g., HOMA-IR) were made at baseline and post-RET. BCAA concentrations were determined by gas-chromatography mass spectrometry (GC-MS). No associations were observed across age with BCAA; however, RET elicited (*p* < 0.05) increases in plasma BCAA (all age-groups), while HOMA-IR scores reduced (*p* < 0.05) following RET. After RET, positive correlations in lean body mass (*p* = 0.007) and strength gains (*p* = 0.001) with fasting BCAA levels were observed. Elevated BCAA are not a robust marker of ageing nor IR in those with a healthy BMI; rather, despite decreasing IR, RET was associated with increased BCAA.

## 1. Introduction

Insulin resistance (IR) is a core pathophysiological mechanism which manifests in concert with β-cell failure, leading to type 2 diabetes mellitus (T2DM) [1] a disease that is expected to increase in prevalence to affect 592 million by 2035 (382 million in 2013) [2]. Given that this disease is most apparent in older individuals (>65 years) [3] in whom life expectancy has increased [4], this has, and will continue, to result in a burgeoning healthcare burden. While the aetiology of IR and T2DM are complex, there has been long-standing curiosity in relation to a potential link between the branched chain amino acids (BCAA) leucine, isoleucine and valine, which account for ~35% of the essential amino acids (EAA) in muscle proteins [4], and IR.

In pioneering studies, Felig et al. [5] demonstrated strong correlations between elevated circulatory BCAA concentrations and insulin levels in human obesity, that were corrected by weight loss. More recently, reports of elevated BCAA in obese and/or IR individuals have been substantiated in longitudinal [6] and cross-sectional [7] studies, as well as across various ethnic groups [8]. For example, Newgard et al. [9] reported positive associations between circulating BCAA concentrations and increased risk of IR and T2DM, and metabolomic profiling of >100 plasma analytes revealed elevated levels of BCAA and select other metabolites as a metabolic “footprint” of IR [9]. This was based on five principle components that accounted for the greatest differences between obese and lean subjects; a combination of BCAA, aromatic AA (phenylalanine and tyrosine), Glx (glutamine and/or glutamate) and acyl-carnitines (C3 and C5), indicating interplay between AA and lipid metabolism [10]. Moreover, the role of BCAA in IR was further cemented with observations that lower BCAA levels correlated with improved markers of insulin sensitivity (IS) following weight-loss interventions enhancing glycaemic control [11,12]. In addition to this, more recent studies have reported that BCAA-mediated IR may be compounded by IR, further exacerbating BCAA accumulation [13,14]. Finally, pre-clinical [7,15,16] and clinical [10,17] studies have shown that BCAA-mediated IR, at least in part, lies at the level of skeletal muscle [11,18].

Nonetheless, that circulatory BCAA positively links to IR does not alter the positive effects that dietary BCAA have upon skeletal muscle metabolism. The role of BCAA in stimulating muscle protein synthesis and supporting muscle hypertrophy following resistance exercise training (RET, [5,19,20]) is well-documented. Further, leucine acts not only as a substrate for newly synthesised proteins and as a regulatory signalling metabolite activating anabolic pathways [21,22], but is also a potent insulin secretagogue with the potential to enhance peripheral glucose uptake and to inhibit whole-body and muscle protein degradation via inducing insulin secretion [23,24,25,26,27]. These properties of BCAA demonstrate the importance of their role in maintaining and increasing skeletal muscle mass [24,25]. Yet despite these key metabolic roles, sustained elevated levels of circulating plasma BCAA remain widely implicated in the pathophysiology of IR [18,19], and the ensuing development of T2DM.

Collectively, previous studies implicate BCAA in the pathogenesis of IR and T2DM. Nonetheless, there remain few intervention-type studies (weight-loss, drugs, exercise, etc.) examining such links under circumstances promoting IS and metabolic health, e.g., exercise. Specifically, RET is one such powerful countermeasure to improve metabolic health and to mitigate age-associated declines in muscle mass and function across the lifespan [28], even in frail elderly individuals [29,30]. As such, we investigated the effects of 20 weeks of fully supervised RET in relation to fasting plasma BCAA concentrations and metabolic/physiological health parameters. We hypothesised that: (1) ageing would be associated with increased BCAA, (2) RET would reduce BCAA and (3) that this would be associated with improvements in IR (i.e., HOMA-IR) and/or or other indices of metabolic health.

## 2. Materials and Methods

### 2.1. Ethical Approval

The present study samples originated from previously published work by our research group [28]. This study was reviewed and approved by the University of Nottingham Faculty of Medicine and Health Sciences Research Ethics Committee (D/2/2006) and complied with the 2013 Declaration of Helsinki. All procedures and risks were thoroughly explained to volunteers and written consent was obtained prior to participation.

### 2.2. Participant Characteristics

Three participant cohorts were studied, consisting of young (18-28 years, *n* = 8, BMI: 24±1 kg/m^2^), middle-aged (45–55 years, *n* = 9, BMI: 27 ± 1 kg/m^2^) and older (65–75 years, *n* = 15, BMI: 27 ± 1 kg/m^2^) men and women (~50:50). All participants were screened by a medical questionnaire (past and existing medical conditions, lifestyle choices), physical examination, clinical chemistry blood profiles (liver function tests (LFTs), thyroid function tests (TFTs), full blood count (FBC), urea and electrolytes (U and E’s), fasting glucose, fasting insulin, clotting factors and lipid profiles) and a resting ECG. Participants were not taking any medication at the time of study, had normal blood chemistry, were normotensive (BP <139/89) and did not smoke (nor had they in the past 5 years). Participants were excluded from the study for any metabolic, respiratory or cardiovascular disorders including insulin resistance, dyslipidaemia, uncontrolled asthma, or family history of heightened cardiovascular disease risk (cardiovascular event <55 years). Participants performed activities of daily living but did not participate in formal aerobic exercise training and had not participated in structured RET in the last 2 years. All study sessions were performed at the University of Nottingham Medical School at the Royal Derby Hospital Centre. The exercise intervention was conducted at two sites, based on geographical proximity to the volunteers. All exercise sessions were fully supervised by a single member of research staff.

### 2.3. Participation Overview

Before study days (before and after RET), volunteers were instructed to refrain from strenuous exercise (including the RET intervention) for 72 h and from alcohol or caffeine for 24 h. On each study day, volunteers reported to the laboratory at 09:00 h, following an overnight fast (water ad libitum) from 21:00 h the evening before. Body composition was assessed via dual-energy X-ray absorptiometry (DXA; Lunar Prodigy II, GE Medical Systems) with all regions automatically assessed by the integrated software package (Encore software, GE Healthcare). Blood samples were taken from the antecubital vein and collected into lithium-heparin containing vacutainers for measures of plasma metabolites, insulin and glucose concentrations, with the plasma-fraction collected following centrifugation at 2000× *g* for 20 min at 4 °C. All samples were stored at −80 °C from collection until further analysis.

The RET programme was designed to achieve muscle hypertrophy based on previous recommendations [31]. As such, the RET programme comprised fully supervised exercise sessions, 3 times each week, with each session lasting approximately 60 min. Two sets of 8–12 repetitions of three upper and three lower body exercises were performed in each session. To achieve progressive overload, training intensity was increased from 40% to 60% 1-RM (repetition maximum) during 4 weeks of induction training (to ensure adoption of correct technique and exercise familiarisation) and was then set at 70% 1-RM for the remainder of the training with 1-RM re-assessment every 4 weeks to ensure progression and consistency of training intensity.

### 2.4. Analytical Methods

#### 2.4.1. Plasma Amino Acid Concentrations

To determine plasma AA concentrations, we added stable isotopically labelled internal standards and prepared samples according to our standard methods [19]. Briefly, heparinized plasma proteins were precipitated with 1 mL ice-cold ethanol and centrifuged at 10,000 rpm for 5 min, the supernatant was removed and evaporated to dryness under nitrogen at 90 °C, followed by re-suspension in 0.5M HCI. Ethyl acetate was then added, and samples were vortexed thoroughly before the upper, ethyl acetate layer (containing lipids) was extracted. The aqueous AA-containing layer was evaporated to dryness under a steady flow of nitrogen at 90 °C. Derivatization of the dry residue was achieved via addition of equal volumes of acetonitrile and N-Methyl-N-(*tert*-butyldimethylsilyl)trifluoroacetamide (MTBSTFA), and incubated at 90 °C for 45 min, thus converting the AA to their *t*-BDMS derivatives [19]. A pooled plasma QC sample was prepared with each batch and injected throughout the batch run to monitor instrument performance over time. This was achieved by pooling small aliquots of each study sample and thoroughly mixing. Aliquots of study-specific samples were used to closely mimic metabolite composition of the samples being tested, with the purpose being to account for analyst and analytical variation during sample preparation and batch run, respectively. AA concentrations were determined with reference to a calibration curve composed of a standard AA mix of known quantity and analysed by GC-MS.

#### 2.4.2. GC-MS Conditions

To quantify plasma AA concentrations, 0.5 µl of sample was injected into an ISQ Trace 1300 single quadrupole GC-MS (ThermoFisher Scientific, Hemel Hempstead, UK). A split injection mode (1:10) was used, at an initial oven temperature of 100 °C held for 1 min, with a temperature ramp of 12 °C/ min to 300 °C and held for 5 min. Helium was used as a carrier gas at a flow rate of 1.5 mL/min, and sample separation was achieved on a 30 m Rxi-5MS (0.25 mm internal diameter, 0.25 µm thickness) fused silica column (Restek, Bellafonte, Pennsylvania). A selected ion monitoring scan (SIM) was created to search AA standards for leucine (mass 302), isoleucine (mass 302) and valine (mass 288), with corresponding isotopically labelled internal standards (304 and 289 for leucine and valine, respectively), or norleucine for isoleucine quantitation, included in the SIM.

### 2.5. Insulin and Glucose Concentrations

Plasma insulin and glucose concentrations, as well as lipoprotein content, was assessed in samples from before and after RET, as reported [28]. In brief, plasma insulin and glucose were measured in duplicate, using undiluted samples. Insulin was assessed via a high-sensitivity human insulin ELISA (DRG Instruments GmbH, Marburg, Germany) according to the manufacturer’s instructions. Plasma glucose was measured using a clinical chemistry analyser (ILAB 300 Plus Clinical Chemistry System, Warrington, Cheshire, UK) against commercial standards. Insulin sensitivity was calculated using the homeostatic model assessment of insulin resistance (HOMA-IR) and the following Formula:

(HOMA-IR = plasma glucose concentration (mmol.l^−1^) × plasma insulin concentration (mU.l^−1^))/22.5

Circulating plasma lipoprotein concentrations (low-density lipoprotein (LDL) and high-density lipoprotein (HDL)) were analysed by the Clinical Pathology Laboratory at the Royal Derby Hospital.

### 2.6. Statistical Analysis

Principal component analysis (PCA) was used as multivariate analysis, firstly to reduce the number of variables to principal component clusters with scores of IS (HOMA-IR) and then with the addition of additional clinical variables (i.e., insulin, glucose, HDL, LDL, etc). Multiple linear regression (MLR) analyses were first used to identify which variables correlated with scores of IS, and then stepwise regression analyses were performed to reveal any potentially novel associations with BCAA concentrations. These correlations were performed at baseline and post-RET. Relationships that were identified were then isolated and further correlation was determined to describe the strength and significance of the interaction. Statistical analysis was performed in R-Studio employing in-house R scripts. Subsequent statistical analyses were confirmed in Prism v8.3 (GraphPad, La Jolla, California, USA) version 7. All data are reported as mean ± SEM, with significance set at *p* < 0.05. Data were tested for normality to determine appropriate analysis. Paired t-tests were used to assess the effects of RET, with Pearson’s correlation used to explore relationships between fasting plasma BCAA concentrations and clinical parameters, such as body composition, fat mass, fat free mass and IS.

## 3. Results

### 3.1. Muscle Mass and Function

The characteristics of our participants at baseline and following RET are listed in Table 1. As previously reported, 20 weeks of our whole-body RET programme elicited improvements in strength irrespective of age. However, whole-body lean mass gains were only seen in the young and middle-aged groups [28], with a significant negative correlation between age and hypertrophy [32].

### 3.2. Circulating BCAA Levels

**Figure 1:**
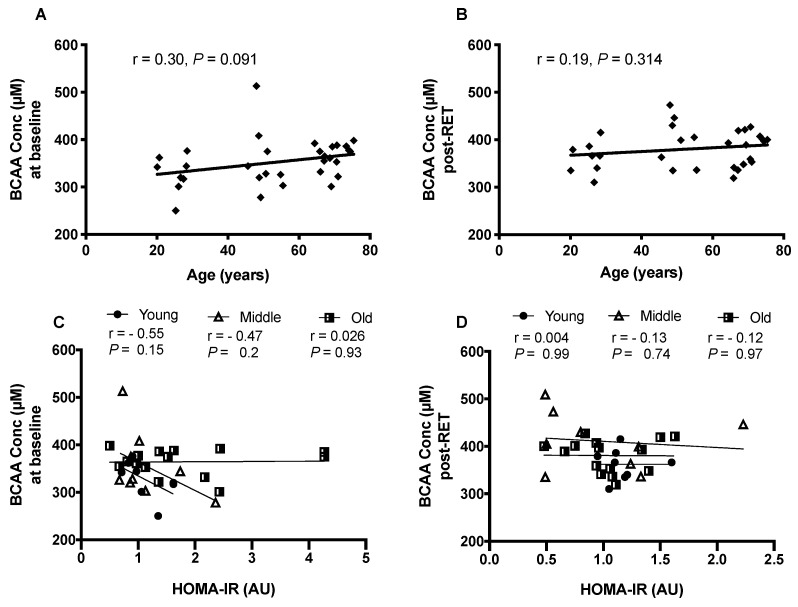
The relationship between circulating BCAA concentrations and age at baseline (**A**) and after (**B**) 20-weeks of supervised, whole-body RET (*n* = 8–15/ group) and in distinct age groups at baseline (**C**) and after RET (**D**).

**Figure 2:**
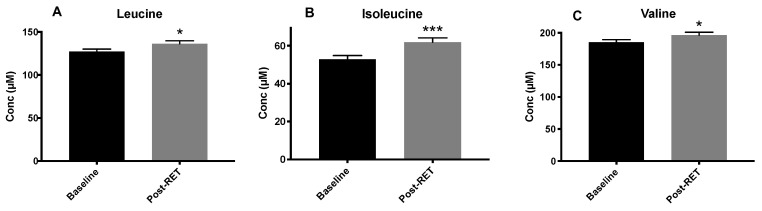
Circulating plasma leucine (**A**), isoleucine (**B**) and valine (**C**) at baseline and after 20-weeks of RET (*n* = 32). Bars represent mean and SEM. Quantification achieved via GC-MS with reference to a calibration curve. Statistical analysis via paired t-tests. * *p* < 0.05; *** *p* < 0.001 vs. baseline.

No correlation was seen with age and BCAA concentrations either pre or post-RET (Figure 1A,B, respectively). Additionally, no relationship existed between HOMA-IR and age either at baseline (1C) or post-RET (1D). For each of the individual age-groups, and when all the age-groups were collapsed into a single cohort, RET resulted in significantly elevated BCAA concentrations (leucine, *p* = 0.0011; isoleucine, *p* = 0.0004; valine, *p* = 0.03 (Figure 2)). Pooled QC throughout the instrumental run yielded ~5% CV for each AA. Furthermore, there were no apparent sex interactions, with both sexes responding similarly to the RET programme. Therefore, data from all age groups were collapsed into one group (*n* = 32).

### 3.3. HOMA-IR and Fasting Plasma BCAA Concentrations

**Figure 3:**
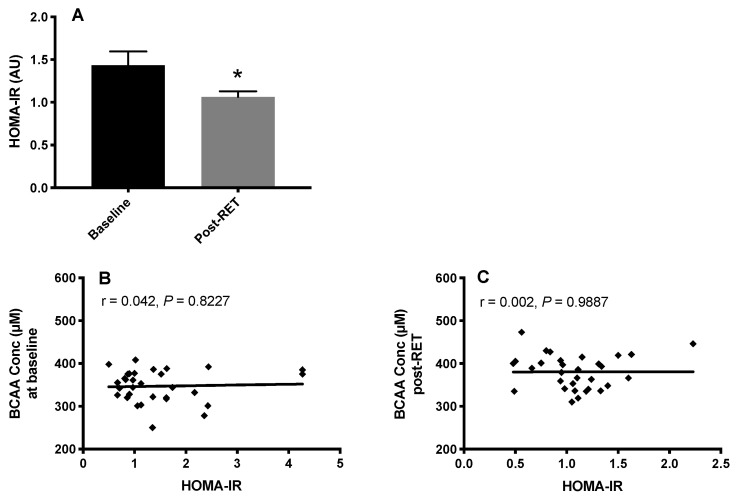
Insulin resistance (via homeostatic model assessment of insulin resistance; HOMA-IR) at baseline and after 20-weeks, whole-body resistance exercise training RET (**A**) and the relationship between IR and circulating BCAA concentrations at baseline (**B**) and after (**C**) RET. * *p* < 0.05 vs. baseline.

Both body mass index (BMI) and HOMA-IR were significantly reduced (*p* < 0.05) after RET, suggesting improved IS in our volunteers (Figure 3A). However, there was no correlation between BCAA concentrations and HOMA-IR in any group either before (Figure 3B) or after RET (Figure 3C), despite significant alterations in each.

### 3.4. Relationships between BCAAs and Clinical Variables of Health

**Figure 4:**
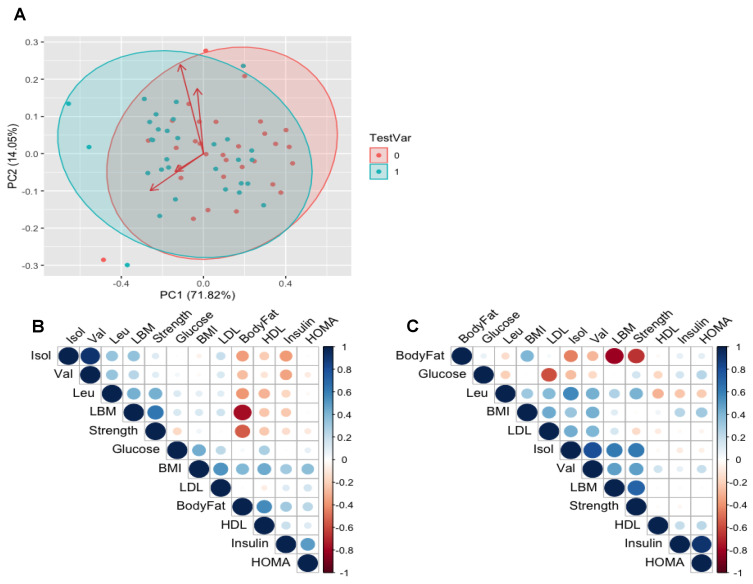
Principle component analysis (PCA) plot visually displaying overlap of clinical variables of health; no distinctive clustering of metabolites predicative of circulating branched chain amino acid (BCAA) concentrations are present at baseline (red), nor emerge following RET (green) (**A**). Heatmap of correlations that are predictive of circulating BCAA concentrations at baseline (**B**) and after RET (**C**). The strength of relationships are based on a scale of −1 (red), representing a negative relationship and 1 (blue) a positive relationship. The strength of the relationships are depicted by the size of the circle.

To visually explore whether there were any novel relationships from our study, data were log transformed for PCA analysis (Figure 4A) to investigate whether there were metabolite clusters which could illustrate differences in variables either at baseline or with RET, however this revealed that there were no distinct clustering of metabolites that co-vary (at baseline or post-RET). Using the same variables and fasting plasma BCAA concentrations, we aimed to explore potential links that may be of interest with a correlational matrix in the form of a heatmap (Figure 4B). MLR was first used to test whether HOMA-IR or other clinical variables of health could predict BCAA concentrations in our healthy participants. At baseline, the results of the linear model predictors explain 29% of the variance with a residual standard error (RSE) of 40.06 on 9 degrees of freedom (DOF) and of 21 (adjusted R^2^ = −0.006, F = 0.978 {on 9 and 21 DOF}, *p* = 0.484). Following RET, the results describe 73% of the variance with a RSE of 30.15 on 21 DOF (adjusted R^2^ = 0.616, F = 0.653 {on 9 and 21 DOF}, *p* = 0.0002) which revealed strength as a significant (*p* = 0.002) associated variable with BCAA and other co-variates. Following this, stepwise regression was used to uncover which combination of our measured co-variates would best predict post-RET levels of plasma BCAA, and the stepwise regression model explains 69% of the variance with an RSE of 28.42 on 27 DOF (adjusted R^2^ = 0.659, F = 20.37 {on 3 and 27 DOF}, *p* = 4.19) which revealed strength (*p* = 0.001), LDL (*p* = 0.001) and BMI (*p* = 0.1) as significant and promising variables in predicting post-RET levels of BCAA.

### 3.5. Muscle Mass, Strength, Body Fat % and Circulating BCAA

**Figure 5:**
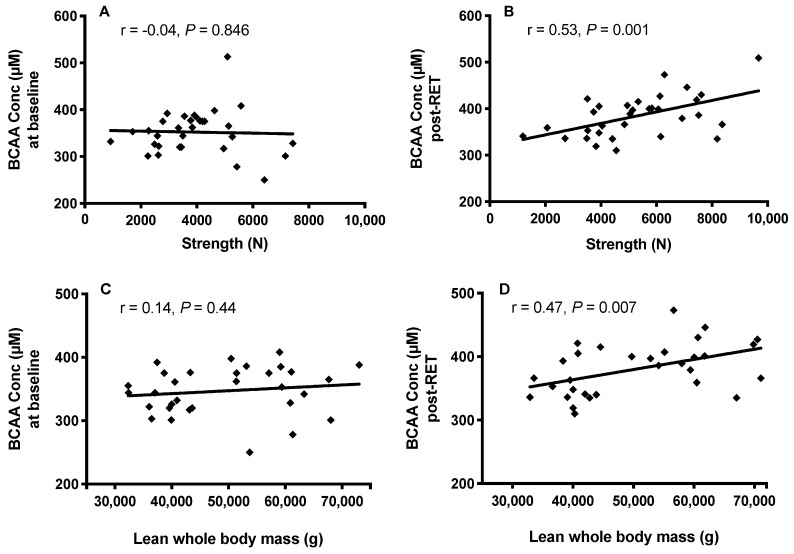
The relationship between circulating branched chain amino acid (BCAA) concentrations and muscle strength (**A**,**B**) and mass (**C**,**D**) at baseline and after 20-weeks, whole-body RET.

Although there was no relationship between plasma BCAA concentrations and muscle strength (r = −0.04, *p* = 0.846) or mass (r = 0.14, *p* = 0.447) prior to RET (Figure 5A,C), increases in muscle strength and mass with RET resulted in significantly positive relationships between BCAA concentrations and both strength (r = 0.53, *p* = 0.001) and mass (r = 0.47, *p* = 0.007) post-RET (Figure 5B,D).

### 3.6. Body Fat, HDL, LDL and Plasma BCAA

**Figure 6:**
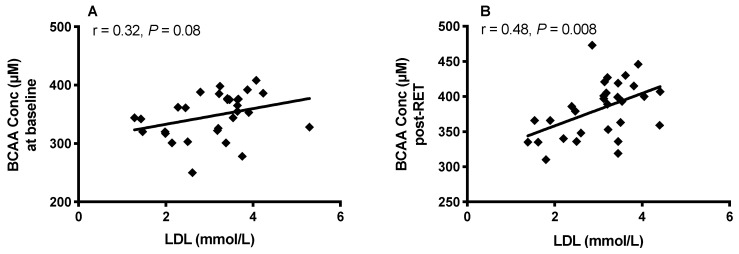
The relationship between circulating BCAA levels and low-density lipoprotein (LDL) before (**A**) and after 20-weeks of RET (**B**).

LDL and HDL levels were unchanged by RET. However, a positive trend between fasting plasma BCAA concentrations and LDL was observed at baseline (r = 0.3, *p* = 0.08; Figure 6A), which was significant following RET (r = 0.48, *p* = 0.008; Figure 6B).

## 4. Discussion

In the present study, we did not observe the commonly reported associations between HOMA-IR and BCAA [10]. These data likely demonstrate that whilst elevated BCAA have been linked to increased risk of T2DM [7] and are a hallmark of obesity [10], this relationship may not hold in individuals within a healthy BMI range. Moreover, our data do point to the notion that elevated BCAA are not an inevitable hallmark of “healthy ageing”. In support of this, in a previous study investigating the links between BCAA and cardio-metabolic risk factors, it was noted that reduced, rather than increased BCAA were evident in very old age [33].

While this may be due to chronic alterations in diet, the ageing gastrointestinal system, and dietary behaviours may have some influence, it once again highlights that elevated fasted plasma BCAA are not an all-encompassing biomarker of metabolic risk. Interestingly, recent studies [34] have shown that BCAAs robustly correlate with HOMA-IR, as well as cardio (metabolic) risk factors and mortality, and so it has been proposed that lowered BCAAs (particularly in older individuals) are inversely associated with health risk factors. This has further been expanded by other studies showing that short-term (28 days) dietary overconsumption increases plasma BCAAs (namely driven by isoleucine and valine) in line with weight gain [35]. However, nutritional intervention studies alone are not able to account for the perturbation in BCAA metabolism which RET can induce, although clearly the link between circulating BCAAs and health continues to remain a paradoxical one. Promisingly, studies have shown that the relationship between plasma BCAAs, dietary intake and their regulation on whole-body weight are comparable in both humans and mice [36], which may provide the potential for future studies examining the role of BCAAs in human health. Although, clearly BCAAs are essential for humans and exercise is beneficial to overall health, therefore caution ought to be taken when proposing whether plasma BCAAs are a positive or negative marker of health, as this relationship is likely to be dependent on context and the demographics of the individuals studied.

It is noted that lean mass gains were not evident in our older participants, and work from our lab shown this may indeed be due to anabolic resistance [37], which itself is multi-faceted (reduced translational capacity, hormonal efficiency and MPS with increased MPB), however RET still remains the most effective method of counteracting the age-related declines in loss of lean mass [38], particularly if it is combined with, for example, exogenous testosterone administration [39] or protein supplementation [40,41].

Based on previous correlations between BCAA and IR, we predicted that RET would improve biomarkers of metabolic risk and concomitantly reduce BCAA concentrations. Instead we noted a reduction in HOMA-IR, in the face of a systematic increase in each of the BCAAs. The lack of this relationship was first highlighted in our principal components’ analyses, and its absence was confirmed with Pearson’s regression. These findings demonstrate that lowering of BCAA is not an inevitable consequence of improved metabolic health, i.e., HOMA-IR. Instead, following RET, our PCA matrix plots comparing a number of variables with plasma BCAA levels revealed the most positively correlated facets to circulatory BCAA to be muscle mass and strength, thus illustrating novel positive links between muscle mass and circulatory BCAA (following RET). Basal (i.e., not under circumstances of exposure to a muscle growth regime) relationships have also been reported; Borg et al. [42] report data on 227 older (>65 y) volunteers from a cross-sectional study showing reduced levels of BCAA correlating with lower skeletal muscle mass, strength and longer sit-to-stand times. These data were also consistent with previous studies [43] supporting the notion that low BCAA concentrations, particularly leucine, correlate with diminishing lean mass and sarcopenia. Our data in healthy individuals are in-line with studies suggesting that BCAA are a marker of muscle mass/strength [42,43], and may also indicate that relationships with obesity could in fact reflect the notion that obese individuals have greater anti-gravity muscle mass than healthy weight counterparts [44,45,46]. In other words, links to fat mass may be a misconception, i.e., with heightened fat mass reflecting heightened lean mass in obese individuals.

An interesting finding of this study was a positive correlation between BCAA and LDL after RET. Increased plasma LDL, particularly in older individuals, is a recognised risk factor for the development of conditions such as metabolic dyslipidaemia and coronary heart disease [47], particularly in the face of reduced HDL levels [48], making this link between elevated BCAA and LDL in the present study an intriguing, though paradoxical, finding given the improvements in other markers of metabolic health. A potential explanation for elevated LDL may be the established link between leucine and valine and cholesterol metabolism. Leucine and valine constituent metabolites (such as α-ketoisocaproate (α-KIC), β-Hydroxy β-methylbutyric acid (HMB), mevalonate and 3-hydroxyisobutyrate (3-HIB)) contribute to an increased cholesterol metabolism [19,49,50,51], which may be the case with our study participants given the increased concentration of BCAA following RET. Increased BCAA concentrations are reported to increase insulin action in hepatic cells [52], resulting in prolonged gluconeogenesis leading to impaired hepatic lipid homeostasis, and subsequent accumulation of triglycerides and other fatty acids [53]. Given that adipose tissue is effective in converting BCAA carbon skeletons to de novo fatty acid synthesis ex-vivo [54] and constitutes a major site where excess BCAA may be converted to lipid species, it would be reasonable to propose that inter-organ metabolism is implicated in the rise of lipid species in plasma. Both liver [52] and adipose tissue [55] may therefore be important organs in defining the relationship between BCAA and dyslipidemia due to their central roles in glucose and lipid metabolism, respectively. Positive associations between plasma BCAA and dyslipidaemia have been reported previously, particularly for circulating LDL [48], and in both diabetic and non-diabetic follow-up studies [15,56]. Moreover, even when adjusted for BMI, BCAA remain significantly correlated to triglyceride levels [56], suggesting at least a partial role of BCAA on circulating lipid species. Alternatively, the changes seen with LDL here could be due in part to the training modality used in the present study because, while the effects of endurance exercise in eliciting reduction of plasma LDL is well-known [57], the effects of RET on the same parameters are not as well established.

The present study is not without its limitations. For example, although our participants are well-matched in terms of lean mass at baseline, our low sample size is an acknowledged limitation, although performing much larger highly controlled interventional trials are clearly a major undertaking. In addition, intra-group variability with regards to daily activity levels may pose potential confounding variables, as high levels of physical activity can lead to inadvertent stimulation of muscle remodelling [58,59]. Also, since our participants were healthy and within a normal BMI range, our results should be extrapolated to cohorts fitting of similar criteria. Additionally, investigation into the effects of dietary intake in both sexes would provide some insight into the regulation of plasma BCAAs; however, absorption rates as well as the quantity of ingested dietary BCAA that eventually reaches blood circulation [60,61,62,63] is unclear, as is whether plasma levels of BCAAs reflect short-term or long-term dietary intake [60]. Rodent studies [64] looking into the long-term effects of dietary BCAA control on health and lifespan have proposed mechanisms that exist for the elevation of BCAA’s which involve notable interactions with tryptophan and threonine. Thus, studies looking into the temporal basis of dietary BCAA intake, and the influence of the gut microbiome [65], could provide an insight into the causal relationships of this link. 

## 5. Conclusions

In summary, twenty weeks RET in a tightly controlled and longitudinal intervention elicits significant increases in all of the BCAA, which are commonly reported to be markers of poor IS. These increases do not correlate with indices of IS. However, they significantly correlate to strength and lean whole-body mass changes (post-RET) irrespective of age or sex, highlighting a novel link that warrants further investigation.

## Figures and Tables

**Table 1:** Participant demographics M: F denotes *n* of males to females per group.

Participant ID	Baseline (BL)	Post-RET
Sex	Young (4: 4 M: F) Middle (5: 4 M: F) Old (8: 7 M: F)	
Age (years)	53 ± 19	
BMI (kg/m^2^)	26 ± 3	26 ± 2
Fasting Glucose (mg/dL)	5.6 ± 0.6	5.3 ± 0.7
Fasting Insulin (µU/mL)	4.9 ± 2	4.5 ± 1.5
HOMA-IR (AU)	1.4 ± 0.9	1.1 ± 0.4

## References

[1] Guariguata L., Whiting D.R., Hambleton I., Beagley J., Linnenkamp U., Shaw J.E. (2014). Global estimates of diabetes prevalence for 2013 and projections for 2035. Diabetes Res. Clin. Pract..

[2] Wild S., Roglic G., Green A., Sicree R., King H. (2004). Global prevalence of Diabetes: Estimates for the year 2000 and projections for 2030. Diabetes Care.

[3] Harper A.E., Miller R.H., Block K.P. (1984). Branched-chain amino acid metabolism. Annu. Rev. Nutr..

[4] Wang H., Naghavi M., Allen C., Barber R.M., Carter A., Casey D.C., Charlson F.J., Chen A.Z., Coates M.M., Coggeshall M. (2016). Global, regional, and national life expectancy, all-cause mortality, and cause-specific mortality for 249 causes of death, 1980–2015: A systematic analysis for the Global Burden of Disease Study 2015. Lancet.

[5] Felig P., Marliss E., Cahill G.F. (1969). Plasma amino acid levels and insulin secretion in obesity. N. Engl. J. Med..

[6] Wang T.J., Larson M.G., Vasan R.S., Cheng S., Rhee E.P., McCabe E., Lewis G.D., Fox C.S., Jacques P.F., Fernandez C. (2011). Metabolite profiles and the risk of developing diabetes. Nat. Med..

[7] Huffman K.M., Shah S.H., Stevens R.D., Bain J.R., Muehlbauer M., Slenz C.A., Tanner C.J., Kuchibhatla M., Houmard J.A., Newgard C.B. (2009). Metabolic Intermediates and Insulin Action in Overweight to Obese, Inactive Men and. Diabetes Care.

[8] Tillin T., Hughes A.D., Wang Q., Würtz P., Ala-Korpela M., Sattar N., Forouhi N.G., Godsland I.F., Eastwood S.V., McKeigue P.M. (2015). Diabetes risk and amino acid profiles: Cross-sectional and prospective analyses of ethnicity, amino acids and diabetes in a South Asian and European cohort from the SABRE (Southall And Brent REvisited) Study. Diabetologia.

[9] Newgard C.B., An J., Bain J.R., Muehlbauer M.J., Stevens R.D., Lien L.F., Haqq A.M., Shah S.H., Arlotto M., Slentz C.A. (2009). A Branched-Chain Amino Acid-Related Metabolic Signature that Differentiates Obese and Lean Humans and Contributes to Insulin Resistance. Cell Metab..

[10] Newgard C.B. (2012). Interplay between lipids and branched-chain amino acids in development of insulin resistance. Cell Metab..

[11] Shah S.H., Crosslin D.R., Haynes C.S., Nelson S., Turer C.B., Stevens R.D., Muehlbauer M.J., Wenner B.R., Bain J.R., Laferrère B. (2012). Branched-chain amino acid levels are associated with improvement in insulin resistance with weight loss. Diabetologia.

[12] Wang Q., Holmes M.V., Smith G.D., Ala-Korpela M. (2017). Genetic support for a causal role of insulin resistance on circulating branched-chain amino acids and inflammation. Diabetes Care.

[13] Mahendran Y., Jonsson A., Have C.T., Allin K.H., Witte D.R., Jørgensen M.E., Grarup N., Pedersen O., Kilpeläinen T.O., Hansen T. (2017). Genetic evidence of a causal effect of insulin resistance on branched-chain amino acid levels. Diabetologia.

[14] Batch B.C., Shah S.H., Newgard C.B., Turer C.B., Haynes C., Bain J.R., Muehlbauer M., Patel M.J., Stevens R.D., Appel L.J. (2013). Branched chain amino acids are novel biomarkers for discrimination of metabolic wellness. Metabolism.

[15] Yamakado M., Nagao K., Imaizumi A., Tani M., Toda A., Tanaka T., Jinzu H., Miyano H., Yamamoto H., Daimon T. (2015). Plasma Free Amino Acid Profiles Predict Four-Year Risk of Developing Diabetes, Metabolic Syndrome, Dyslipidemia, and Hypertension in Japanese Population. Sci. Rep..

[16] Wurtz P., Soininen P., Kangas A.J., Rönnemaa T., Lehtimäki T., Kähönen M., Viikari J.S., Raitakari O.T., Ala-Korpela M. (2013). Branched-chain and aromatic amino acids are predictors of insulin resistance in young adults. Diabetes Care.

[17] Neinast M.D., Jang C., Hui S., Murashige D.S., Chu Q., Morscher R.J., Li X., Zhan L., White E., Anthony T.G. (2018). Quantitative Analysis of the Whole-Body Metabolic Fate of Branched-Chain Amino Acids. Cell Metab..

[18] Smith K., Barua J.M., Watt P.W., Scrimgeour C.M., Rennie J. (1992). Flooding with L- [ IJ3C ] leucine stimulates human muscle protein incorporation of continuously infused L- [ F3C ] valine. Am. J. Physiol. Endocrinol. Metab..

[19] Wilkinson D.J., Hossain T., Hill D.S., Phillips B.E., Crossland H., Williams J., Loughna P., Churchward-Venne T.A., Breen L., Phillips S.M. (2013). Effects of leucine and its metabolite β-hydroxy-β-methylbutyrate on human skeletal muscle protein metabolism. J. Physiol..

[20] Kumar V., Selby A., Rankin D., Patel R., Atherton P., Hildebrandt W., Williams J., Smith K., Seynnes O., Hiscock N. (2009). Age-related differences in the dose-response relationship of muscle protein synthesis to resistance exercise in young and old men. J. Physiol..

[21] Drummond M.J., Fry C.S., Glynn E.L., Dreyer H.C., Dhanani S., Timmerman K.L., Volpi E., Rasmussen B.B. (2009). Rapamycin administration in humans blocks the contraction-induced increase in skeletal muscle protein synthesis. J. Physiol..

[22] Flakoll P.J., Brown L., Hill J., Abumrad N., Paul J., Kulaylat M., Frexes- M., Hourani H., Brown L.L. (1989). Amino acids augment insulin ’s of whole body proteolysis. Am. J. Physiol. Endocrinol. Metab..

[23] Fryburg D.A., Barrett E.J., Louard R.J., Gelfand R.A., David A., Barrett E.J., Louard R.J. (1990). Effect of starvation on human muscle protein metabolism and its response to insulin. Am. J. Physiol. Endocrinol. Metab..

[24] Petrides A.S., Luzi L., DeFronzo R.A. (1994). Time-dependent regulation by insulin metabolism in young healthy adults. Am. J. Physiol. Endocrinol. Metab..

[25] Moller-Loswick A.C., Zachrisson H., Hyltander A., Körner U., Matthews D.E., Lundholm K. (1994). Insulin selectively attenuates breakdown of nonmyofibrillar proteins in peripheral tissues of normal men. Am. J. Physiol. Endocrinol. Metab..

[26] Chow L.S., Albright R.C., Bigelow M.L., Toffolo G., Cobelli C., Nair K.S., Lisa S., Albright R.C., Bigelow M.L., Cobelli C. (2006). Mechanism of insulin’s anabolic effect on muscle: Measurements of muscle protein synthesis and breakdown using aminoacyl-tRNA and other surrogate measures. Am. J. Physiol. Endocrinol. Metab..

[27] Lynch C.J., Adams S.H. (2014). Banched-chain amino acids in metabolic signalling and Insulin resistance. Nat. Rev. Endocrinol..

[28] Phillips B.E., Williams J.P., Greenhaff P.L., Smith K., Atherton P.J. (2017). Physiological adaptations to resistance exercise as a function of age. JCI Insight.

[29] Yarasheski K.E., Pak-loduca J., Hasten D.L., Obert K.A., Brown B., Sinacore D.R., Trappe T.A., Carroll C.C., Dickinson J.M., Lemoine J.K. (1999). Resistance exercise training increases mixed muscle protein synthesis rate in frail women and men ≥ 76 yr old exercise in older adults older men. Am. J. Physiol..

[30] Binder E., Yarasheski K., Steger-May K., Sinacore D.R., Brown M., Schechtman K., Holloszy J. (2005). Effects of progressive resistance training on body composition in frail older adults: Results of a randomized, controlled trial. J. Gerontol..

[31] Singh M.A.F. (2002). Exercise comes of age: Rationale and recommendations for a geriatric exercise prescription. J. Gerontol. Ser. A Biol. Sci. Med. Sci..

[32] Phillips B., Williams J., Atherton P., Smith K., Hildebrandt W., Rankin D., Greenhaff P., MacDonald I., Rennie M.J. (2012). Resistance exercise training improves age-related declines in leg vascular conductance and rejuvenates acute leg blood flow responses to feeding and exercise. J. Appl. Physiol..

[33] Sun L., Hu C., Yang R., Lv Y., Yuan H., Liang Q., He B., Pang G., Jiang M., Dong J. (2017). Association of circulating branched-chain amino acids with cardiometabolic traits differs between adults and the oldest-old. Oncotarget.

[34] Le Couteur D.G., Ribeiro R., Senior A., Hsu B., Hirani V., Blyth F.M., Waite L.M., Simpson S.J., Naganathan V., Cumming R.G. (2020). Branched Chain Amino Acids, Cardiometabolic Risk Factors and Outcomes in Older Men: The Concord Health and Ageing in Men Project. J. Gerontol. Ser. A.

[35] Elshorbagy A.K., Samocha-Bonet D., Jernerén F., Turner C., Refsum H., Heilbronn L.K. (2018). Food Overconsumption in Healthy Adults Triggers Early and Sustained Increases in Serum Branched-Chain Amino Acids and Changes in Cysteine Linked to Fat Gain. J. Nutr..

[36] Ribeiro R.V., Solon-Biet S.M., Pulpitel T., Senior A.M., Cogger V.C., Clark X., O’Sullivan J., Koay Y.C., Hirani V., Blyth F.M. (2019). Of Older Mice and Men: Branched-Chain Amino Acids and Body Composition. Nutrients..

[37] Brook M.S., Wilkinson D.J., Mitchell W.K., Lund J.N., Phillips B.E., Szewczyk N.J., Greenhaff P.L., Smith K., Atherton P.J. (2016). Synchronous deficits in cumulative muscle protein synthesis and ribosomal biogenesis underlie age-related anabolic resistance to exercise in humans Key points. J. Physiol..

[38] Peterson M.D., Sen A., Gordon P.M. (2011). Influence of resistance exercise on lean body mass in aging adults: A meta-analysis. Med. Sci. Sports Exerc..

[39] Gharahdaghi N., Rudrappa S., Brook M.S., Idris I., Crossland H., Hamrock C., Abdul Aziz M.H., Kadi F., Tarum J., Greenhaff P.L. (2019). Testosterone therapy induces molecular programming augmenting physiological adaptations to resistance exercise in older men. J. Cachexia Sarcopenia Muscle.

[40] Esmarck B., Andersen J.L., Olsen S., Richter E.A., Mizuno M., Kjær M. (2001). Timing of postexercise protein intake is important for muscle hypertrophy with resistance training in elderly humans. J. Physiol..

[41] Tieland M., Dirks M.L., van der Zwaluw N., Verdijk L.B., van de Rest O., de Groot L.C.P.G.M., van Loon L.J.C. (2012). Protein Supplementation Increases Muscle Mass Gain During Prolonged Resistance-Type Exercise Training in Frail Elderly People: A Randomized, Double-Blind, Placebo-Controlled Trial. J. Am. Med. Dir. Assoc..

[42] Borg T.S., Luiking Y.C., van Helvoort A., Boirie Y., Schols J.M.G.A., de Groot C.P.G.M. (2019). Low Levels of Branched Chain Amino Acids, Eicosapentaenoic Acid and Micronutrients are Associated with Low Muscle Mass, Strength and Function in Community-Dwelling Older Adults. J. Nutr. Health Aging.

[43] Mcdonald C.K., Ankarfeldt M.Z., Capra S., Bauer J., Raymond K., Heitmann B.L. (2016). Lean body mass change over 6 years is associated with dietary leucine intake in an older Danish population. Br. J. Nutr..

[44] Bosco C., Rusko H., Hirvonen J. (1986). The efffect of extra-load conditioning on muscle performance in athletes. Med. Sci. Sports Exerc..

[45] Hulens M., Vansant G., Lysens R., Claessens A.L., Muls E., Brumagne S. (2001). Study of differences in peripheral muscle strength of lean versus obese women: An allometric approach. Int. J. Obes..

[46] Maffiuletti N.A., Jubeau M., Munzinger U., Bizzini M., Agosti F., De Col A., Lafortuna C.L., Sartorio A. (2007). Differences in quadriceps muscle strength and fatigue between lean and obese subjects. Eur. J. Appl. Physiol..

[47] Seals D.R., Allen W.K., Hurley B.F., Dalsky G.P., Ehsani A.A., Hagberg J.M. (1984). Elevated high-density lipoprotein cholesterol levels in older endurance athletes. Am. J. Cardiol..

[48] Fukushima K., Harada S., Takeuchi A., Kurihara A., Iida M., Fukai K., Kuwabara K., Kato S., Matsumoto M., Hirata A. (2019). Association between dyslipidemia and plasma levels of branched-chain amino acids in the Japanese population without diabetes mellitus. J. Clin. Lipidol..

[49] Nissen S.L., Abumrad N. (1997). Nutritional role of the leucine metabolite p=hydroxy p-methylbutyrate (HMB). Nutr. Biochem..

[50] Duan Y., Li F., Li Y., Tang Y., Kong X. (2016). The role of leucine and its metabolites in protein and energy metabolism. Amino Acids.

[51] Rudney H., Ferguson J.J. (1957). The biosynthesis of 8-hydroxy-3-methylglutaryl coenzyme A. J. Am. Chem. Soc..

[52] Xiao F., Huang Z., Li H., Yu J., Wang C., Chen S., Meng Q., Cheng Y., Gao X., Li J. (2011). Leucine deprivation increases hepatic insulin sensitivity via GCN2/mTOR/S6K1 and AMPK pathways. Diabetes.

[53] Donnelly K.L., Smith C.I., Schwarzenberg S.J., Jessurun J., Boldt M.D., Parks E.J. (2005). Sources of fatty acids stored in liver and secreted via lipoproteins in patients with nonalcoholic fatty liver disease. J. Clin. Investig..

[54] Rosenthal J., Angel A., Farkas J. (1974). Metabolic fate of leucine: A significant sterol precursor in adipose tissue and muscle. Am. J. Physiol..

[55] Herman M.A., She P., Peroni O.D., Lynch C.J., Kahn B.B., Deaconess B.I., State T.P. (2010). Adipose tissue branched chain amino acid (BCAA) metabolism modulates circulating BCAA levels. J. Biol. Chem..

[56] Mook-Kanamori D.O., Römisch-Margl W., Kastenmüller G., Prehn C., Petersen A.K., Illig T., Gieger C., Wang-Sattler R., Meisinger C., Peters A. (2014). Increased amino acids levels and the risk of developing of hypertriglyceridemia in a 7-year follow-up. J. Endocrinol. Investig..

[57] Halverstadt A., Phares D.A., Wilund K.R., Goldberg A.P., Hagberg J.M. (2007). Endurance exercise training raises high-density lipoprotein cholesterol and lowers small low-density lipoprotein and very low-density lipoprotein independent of body fat phenotypes in older men and women. Metabolism.

[58] Aoyagi Y., Shephard R.J. (2013). Sex differences in relationships between habitual physical activity and health in the elderly: Practical implications for epidemiologists based on pedometer/accelerometer data from the Nakanojo Study. Arch. Gerontol. Geriatr..

[59] DiPietro L. (2001). Physical Activity in Aging: Changes in Patterns and Their Relationship to Health and Function. J. Gerontol..

[60] Rietman A., Schwarz J., Tomé D., Kok F.J., Mensink M. (2014). High dietary protein intake, reducing or eliciting insulin resistance?. Eur. J. Clin. Nutr..

[61] Cortiella J., Matthews D.E., Hoerr R.A., Bier D.M., Young V.R. (1988). Leucine kinetics at graded intakes in young men: Quantitative fate of dietary leucine. Am. J. Clin. Nutr..

[62] Biolo G., Tessari P., Inchiostro S., Bruttomesso D., Fongher C., Sabadin L., Fratton M.G., Valerio A., Tiengo A. (1992). Leucine and phenylalanine kinetics during mixed meal ingestion: A multiple tracer approach. Am. J. Physiol. Endocrinol. Metab..

[63] Meguid M.M., Matthews D.E., Bier D.M., Meredith C.N., Young V.R. (1986). Valine kinetics at graded valine intakes in young men. Am. J. Clin. Nutr..

[64] Solon-Biet S.M., Cogger V.C., Pulpitel T., Wahl D., Clark X., Bagley E.E., Gregoriou G.C., Senior A.M., Wang Q.P., Brandon A.E. (2019). Branched-chain amino acids impact health and lifespan indirectly via amino acid balance and appetite control. Nat. Metab..

[65] Meslier V., Laiola M., Roager H.M., De Filippis F., De Filippis F., Roume H., Quinquis B., Giacco R., Mennella I., Ferracane R. (2020). Mediterranean diet intervention in overweight and obese subjects lowers plasma cholesterol and causes changes in the gut microbiome and metabolome independently of energy intake. Gut.

